# Tree-Based Risk Factor Identification and Stroke Level Prediction in Stroke Cohort Study

**DOI:** 10.1155/2023/7352191

**Published:** 2023-04-10

**Authors:** Junyao Li, Yuxiang Luo, Meina Dong, Yating Liang, Xuejing Zhao, Yafeng Zhang, Zhaoming Ge

**Affiliations:** ^1^School of Mathematics and Statistics, Center for Data Science, Lanzhou University, Lanzhou, 730000, China; ^2^Stroke Center, Lanzhou University Second Hospital, Lanzhou, 730030, China

## Abstract

*Objective*. This study focuses on the identification of risk factors, classification of stroke level, and evaluation of the importance and interactions of various patient characteristics using cohort data from the Second Hospital of Lanzhou University. *Methodology*. Risk factors are identified by evaluation of the relationships between factors and response, as well as by ranking the importance of characteristics. Then, after discarding negligible factors, some well-known multicategorical classification algorithms are used to predict the level of stroke. In addition, using the Shapley additive explanation method (SHAP), factors with positive and negative effects are identified, and some important interactions for classifying the level of stroke are proposed. A waterfall plot for a specific patient is presented and used to determine the risk degree of that patient. *Results and Conclusion*. The results show that (1) the most important risk factors for stroke are hypertension, history of transient ischemia, and history of stroke; age and gender have a negligible impact. (2) The XGBoost model shows the best performance in predicting stroke risk; it also gives a ranking of risk factors based on their impact. (3) A combination of SHAP and XGBoost can be used to identify positive and negative factors and their interactions in stroke prediction, thereby providing helpful guidance for diagnosis.

## 1. Introduction

Stroke is an acute cerebral vascular disease that is mainly caused by sudden cerebral vascular rupture or blockage of blood vessels (termed ischemic and hemorrhagic stroke, respectively), leading to brain tissue damage. Stroke has high morbidity, mortality, and disability rates. Ischemic stroke accounts for 60–70% of the total incidence stroke; however, hemorrhagic stroke has a higher mortality rate.

Extensive research has focused on determining the premonitory signs of stroke. The Framingham study [1] reported a series of risk factors for stroke, including age, systolic blood pressure, antihypertensive therapy, diabetes, smoking, previous cardiovascular disease, atrial fibrillation, and left ventricular hypertrophy based on electrocardiogram. Recently, many other studies have found additional risk factors, including creatinine levels and time taken to walk 15 feet [2, 3]. Medical data sets tend to contain large numbers of features; thus, it is a time-consuming task to manually identify and verify risk factors using the available data. However, machine learning methods can effectively identify features that are strongly related to the incidence of stroke based on a large number of feature sets [4]. Therefore, machine learning can be used to improve the accuracy of stroke risk prediction and discover new risk factors.

Models for prediction models of stroke have also been extensively studied. [2] developed a 5-year stroke prediction model based on a cardiovascular health research data set. Machine learning algorithms have also been widely explored in this field, for instance, to predict outcomes of patients with ischemic stroke after intra-arterial therapy using clinical variables [5] and those of patients with brain arteriovenous malformations after endovascular treatment [6]. Among other methods, logistic regression and random forest have shown good performance in predicting the daily activities of discharged patients [7]. Deep learning algorithms that use computed tomography and magnetic resonance imaging features together with clinical variables have been developed to predict hemorrhagic transformation after intravascular therapy [8], visual field defect improvements [9], and speech and motor outcomes [10, 11].

The interpretation of the results of machine/deep learning models has crucial importance in medical applications. In the past few years, machine learning has been used to improve cancer diagnosis, detection, prediction, and prognosis; however, studies usually regard machine learning as a “black box” [12], which limits the confidence of patients and clinicians in the predictions of the models. [13] proposed the use of Shapley additive explanation (SHAP) to elucidate machine learning predictions based on game theory. They have introduced several versions of SHAP (e.g., DeepSHAP, KernelSHAP, LinearSHAP, and TreeSHAP) for specific machine learning model categories. In this study, we interpret machine learning based on TreeSHAP [14–16] to judge the impact of a single feature on different stroke levels and the outcomes of individual cases and to explain the predictions of the machine learning method. Numerous machine-learning-based models have been applied to categorical data and have shown great promise. However, because of the ordering of the response variables in records of stroke level, it is necessary to adapt a traditional classification model to ordinal variables. The most common models are so-called cumulative logit or probit models; these can be specified as logit or probit models for the probabilities of exceeding each of the ordered categories (except the last) [17]. Alternatively, some researchers have integrated the results of modeling research by treating ordered variables as continuous variables or “special” variables in an attempt to provide guidance to researchers [18, 19]. Numerous methods have been proposed to improve stroke prediction; however, most of the relevant studies have focused on the probability of death, dementia, or institutionalization over a fixed number of years. For instance, [20] weighted the modified Rankin scale (mRs) in ordinal analyses for stroke and other neurological disorders, as state transitions differ in clinical prognosis; and [21] assessed the distribution of mRs scores across different strata in AIS according to usual eligibility criteria.

This study focuses on the application of machine learning methods to survey data, where stroke levels are presented as ordinal variables from 0 to 4. The main contribution of this study is to extend the traditional binary/multiclassification to the cumulative binary classifier of *Y* ≥ *k* vs. *Y* < *k* (for all possible *k*) to construct a multiclassifier for ordinal responses. We focus on the identification of the main risk factors for stroke and the prediction of stroke level based on these risk factors. We also consider the effects of risk factors in individual patients, including interaction effects. Risk factors are identified from the cohort data based primarily on Pearson correlation and a mutual information measure; then, stroke level is predicted using a well-known multicategorical classification model. A SHAP-based interpretation is also used to provide a detailed explanation of each factor in an individual diagnosis.

The remainder of the paper is organized as follows.  Section 2 describes the exploration of the stroke data and risk factor identification based on Pearson correlation and the mutual information criterion.  Section 3 presents the prediction of stroke level based on multicategorical classifiers. The model's interpretation with respect to feature importance, positive and negative effects, and interactions, as well as personal prediction and treatment, is presented in Section 4.  Section 5 gives our conclusion and some discussion.

## 2. Exploration of the Stroke Data

The stroke data set was from the Stroke Center, Lanzhou University Second Hospital, from 2016 to 2018, and was part of a national stroke screening project. The questionnaires were designed and administered by the Chinese National Stroke Center of Lanzhou University Second Hospital each year to detect cardiovascular disease risk factors for people over 35 years old in Gansu Province of China. The data set consisted of 12391 samples with 20 variables. After removing seven private personal characteristics that were obviously not related to stroke level, 12 predictors remained: age, gender, history of stroke, history of transient ischemia, family history of stroke, atrial fibrillation or valvular heart disease, hypertension, dyslipidemia, diabetes, smoking history, apparent overweight or obesity, and lack of exercise. The sample consisted of 276 cases of transient ischemic attack (TIA), 9010 low-risk individuals, 1370 of medium-risk individuals, 1617 high-risk individuals, and 118 stroke cases.

Details of the data are provided in Table 1. Note that for categorical features with two options, the 0-1 encoding method was adopted, and the level of stroke (*Y*) was represented as an ordinal variable: 0 (TIA), 1 (low risk), 2 (medium risk), 3 (high risk), or 4 (stroke).

Table 1 also shows the results of five-sample testing of the differences among groups using analysis of variance. *P* values less than 0.01 were observed for all characteristics, indicating that the scores for all factors were statistically significant in classification of stroke level.

The linear relationship and nonlinear dependent relationships among the various factors (*X*_*i*_) and stroke level (*Y*) were studied using Spearman correlation and normal-mutual information (NMI). The results of these analyzes for data 2016 to 2018 are shown in Table 2. Age (*X*_1_) and gender (*X*_2_) had small NMI and Spearman correlation values, indicating that these factors can be discarded because of their weak relationships with stroke level. The most important factors associated with stroke level were hypertension, diabetes, family history of stroke, history of transient ischemia, and lack of exercise.

Hereafter, in this paper, the factors of age and gender are discarded from consideration in the prediction and interpretation procedure.

## 3. Prediction of Stroke Level Based on Multicategorical Classifiers

Risk factors for stroke were primarily identified based on machine learning; then, stroke level was predicted using classifiers.

### 3.1. Multicategorical Classifiers for the Prediction of Stroke Level

Four multicategorical classifiers were used to predict the level of stroke.

#### 3.1.1. Multiple Logistic Regression

Multiple logistic regression is an extension of the binomial logistic regression model for multiple classification and is used to predict the probabilities of different possible outcomes for a category distribution of dependent variables. Specifically, a probability model is used to calculate the probability of obtaining a certain result in the predicted dependent variable after the linear combination of independent variables and corresponding parameters.

#### 3.1.2. Multiple Classification Support Vector Machine

The multiple classification support vector machine (MCSVM) is mainly used for the construction of multiclassifiers by combining many binary classifiers. The one-versus-one method and one-versus-rest method are commonly used. In this study, the small-against-large (*Y* ≤ *k* vs. *Y* > *k*) method is used to predict levels of stroke.

#### 3.1.3. XGBoost

XGBoost, or “extreme gradient boosting,” is a type of boosting ensemble algorithm, which represents an improvement of the gradient boosting decision tree (GBDT) algorithm. The XGBoost algorithm adds regularization to the objective function. When the base learner is CART, the regularization is related to the number of leaf nodes of the tree and the values of the leaf node.

#### 3.1.4. Light Gradient Boosting Machine

The light gradient boosting machine (LightGBM) is a type of boosting integrated algorithm; it is also an efficient implementation of the GBDT algorithm. It first uses a histogram algorithm to transform a traversal sample into a traversal histogram to reduce time complexity. Then, a gradient-based one-side sampling algorithm is used to filter out samples with small gradient in the training process to reduce the computation time. Moreover, a leaf-wise algorithm-based growth strategy is used to construct trees to reduce unnecessary overhead.

Concerning the ordinal response, all the classification algorithms were modified such that they could handle ordinal variables. Specifically, the ordinal responses were partitioned into two categories (*Y* ≤ *k* vs. *Y* > *k* for each possible *k*); then, all classifiers were applied to these binary categories.

### 3.2. Performance of the Multicategorical Classifiers

The data were divided into five mutually exclusive sets by pooling, and classification performance was evaluated by fivefold cross-validation with stratified XGBoost sampling with respect to area under the curve (AUC), accuracy, *F*_1_, recall, and precision.

The results of the evaluation of model performance are shown in Table 3. All four models achieved acceptable results for classification, with AUC > 0.98, for example, whereas LightGBM and XGBoost showed better accuracy (above 0.9) compared with the others. The evaluation indicators of XGBoost were almost the best. Besides, owing to its capacity for interpretation, XGBoost is the preferred model for many applications.

## 4. Model Interpretation Based on SHAP for XGBoost Algorithm

The interpretation of the results of machine-learning-based models has a crucial role in medical research and clinical applications. In this work, SHAP [13] measurements based on the best machine learning model (XGBoost) are used for explanatory data analysis. This further illustrates the effectiveness of the algorithm proposed in this paper and provides guidance for the practical use of the model in diagnosis and survival analysis.

SHAP is a package of interpreted models that can be constructed and used to interpret any machine learning model. It originates from cooperative game theory, where each of its features can be seen as a contributor. When a value is predicted for any sample and the corresponding predicted value is obtained, the SHAP value is called the predicted value of any feature in this sample.

### 4.1. Feature Importance Evaluation

**Figure 1:**
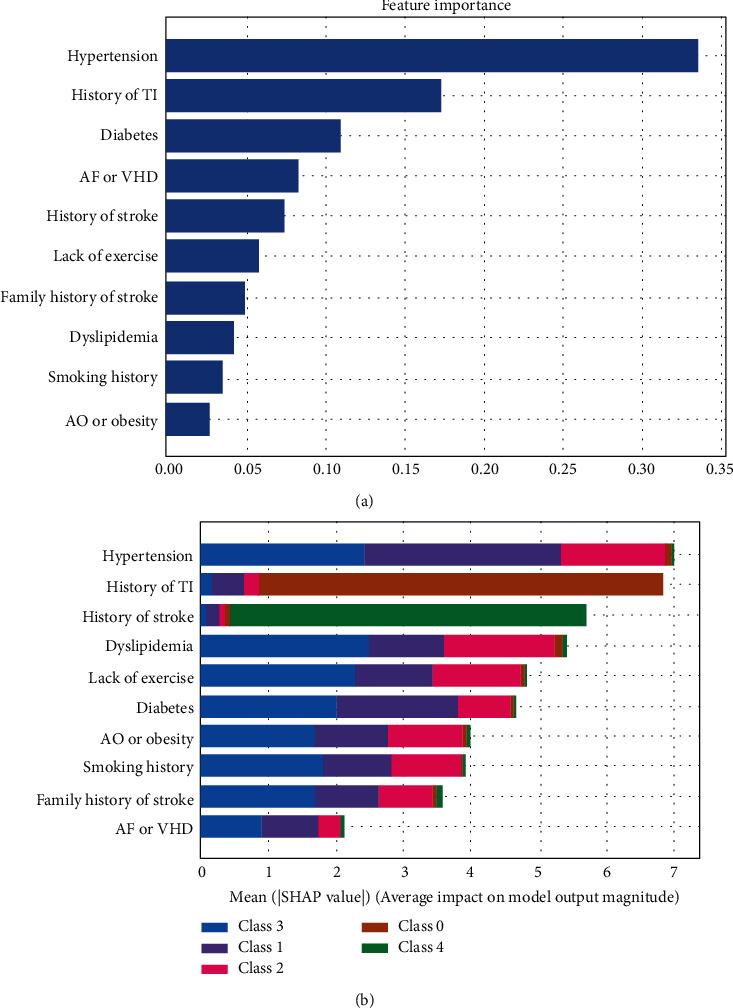
Feature importance of factors based on (a) XGBoost and (b) SHAP.

Figure 1 gives the feature importance rankings of this model evaluated by XGBoost and SHAP. As shown in Figure 1(a), hypertension was the most important factor in the evaluation of stroke, followed by history of transient ischemia, diabetes, atrial fibrillation or valvular heart disease, and history of stroke. The SHAP-based description shown in Figure 1(b) gives a more accurate view of each factor's effect; hypertension, history of transient ischemia, history of stroke, and diabetes are still the most important features, consistent with the results obtained with XGBoost.

From the results shown in Figure 1(b), we can conclude the following.

Hypertension is the most important factor at all stages of stroke, although it has less effect in the case of TIA (class 0).

History of TIA is almost the characteristic of the TIA, and history of stroke is the conclusive factor for recognizing stroke (class 4).

The other factors have significant impact in all stages of stroke.

### 4.2. Evaluation of Individual Features in Stroke Level Prediction

**Figure 2:**
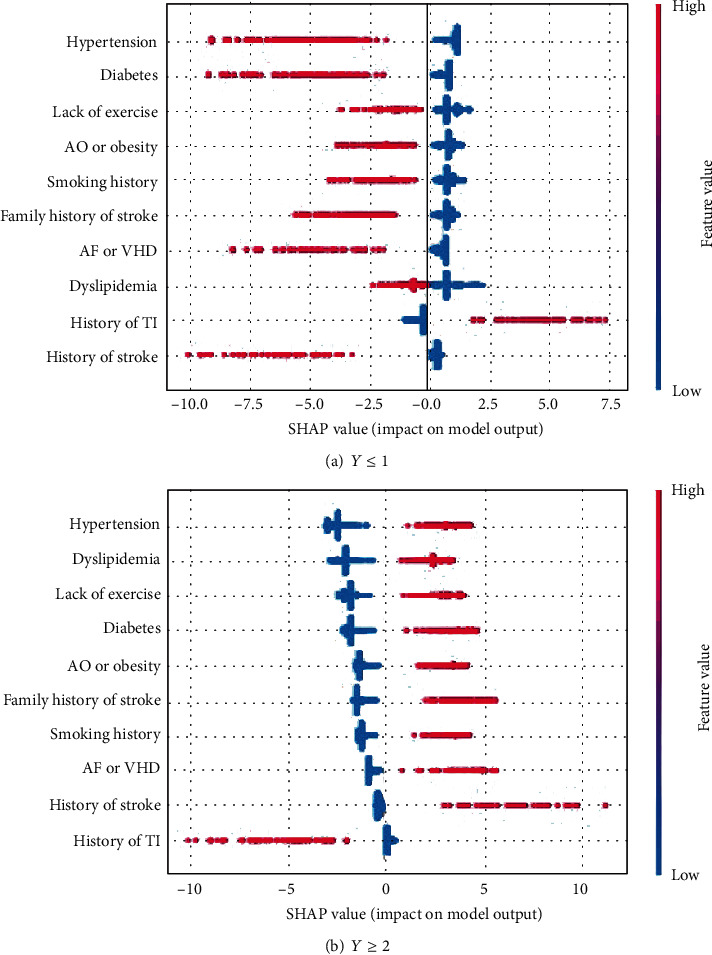
SHAP values for feature importance.

To better understand the specific impact of individual features on different degrees of stroke, overall SHAP feature plots are constructed and are shown in Figure 2 (here, only the cases *Y* ≤ 1 and *Y* ≥ 2 are presented). All factors are listed on the vertical axis ranked by importance. For a specified factor, each point indicates a patient to whom that factor applies (in red) or does not apply (in blue). Right side of a patient in red means it has the positive impact for lying in the corresponding level.

A SHAP description for patients in the high-risk category is shown in Figure 2. It shows that patients with a history of transient ischemia or history of stroke are not likely be classified in the higher-risk stroke subgroup (*Y* > 1) (in fact, history of stroke is the most important identified factor for the occurrence of stroke, and a patient who has experienced TIA before is more likely to be categorized into class 0 (TIA)), whereas the other factors have a strong positive impact, meaning that a patient with the corresponding phenotypes is more likely to be classified as at higher risk of stroke. Similarly, a patient with TIA cannot be classified in the higher-risk category (*Y* ≥ 2).

The same conclusion can be obtained for dyslipidemia, diabetes, lack of exercise, and atrial fibrillation or valvular heart disease. In addition, a SHAP value near 0 means that the corresponding factor makes a small contribution to the development of stroke. Similarly, the negative SHAP values for history of stroke (in red), obesity, family history of stroke, and history mean the stroke level cannot be low risk or TIA.

### 4.3. Interaction Effects for Stroke Level Prediction

**Figure 3:**
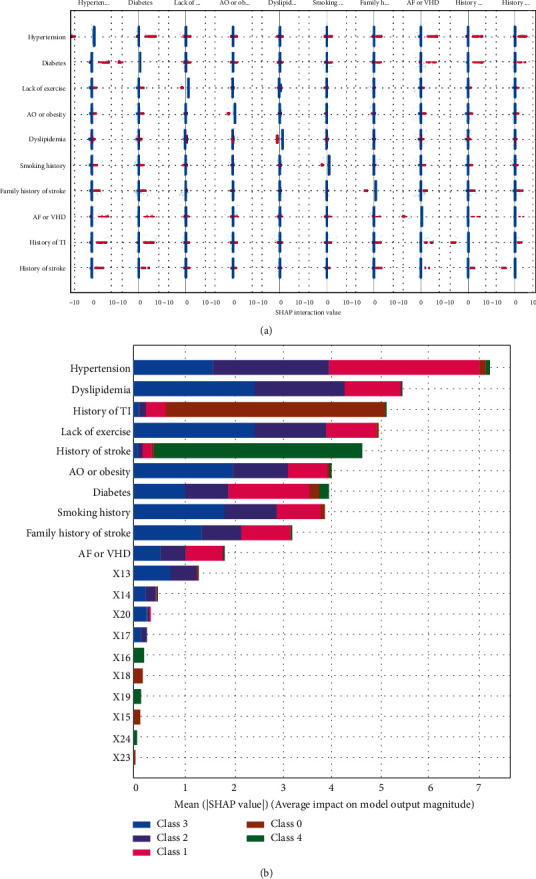
Interaction diagrams of all features at five stroke levels.

The interaction values shown in Figure 3(a) in the low-risk case indicate that although the individual factors have negative influences, the following interactions have strong positive influence:

1. Hypertension and diabetes (the interaction value is recorded as *X*_13_), hypertension and AF/VHD (*X*_14_), hypertension and history of TI (*X*_15_), and hypertension and history of stroke (*X*_16_)
2. Diabetes and AF/VHD (*X*_17_), diabetes and history of TI (*X*_18_), and diabetes and history of stroke (*X*_19_)
3. Family history of stroke and hypertension (*X*_20_), family history of stroke and diabetes (*X*_21_), family history of stroke and AF/VHD (*X*_22_), family history of stroke and history of TI (*X*_23_), and family history of stroke and stroke (*X*_24_)

Similar interactions can be found for other categorical factors in stroke risk level.  Figure 3(b) again gives the importance of the factors; compared with the effect of a single factor, most of the interactions are negligible, except that of hypertension and diabetes.

In addition, we put the interaction values into the machine learning model; the AUCs are shown in Table 4, using the forward stepwise method to add to the original model. After adding the *X*_13_ variable, the accuracy of the model showed a marked improvement. When this variable was added to *X*_17_, the model accuracy reached almost 1, so the procedure can be ended from the addition of *X*_17_. The interaction values *X*_13_, *X*_14_, *X*_20_, and *X*_17_ play a greater part in promoting the occurrence of different degrees of stroke compared with other interaction values. This knowledge is crucial for medical research and clinical applications, and it provides a better theoretical basis for treatment of patients.

### 4.4. Individual Precision Prediction and Treatment

**Figure 4:**
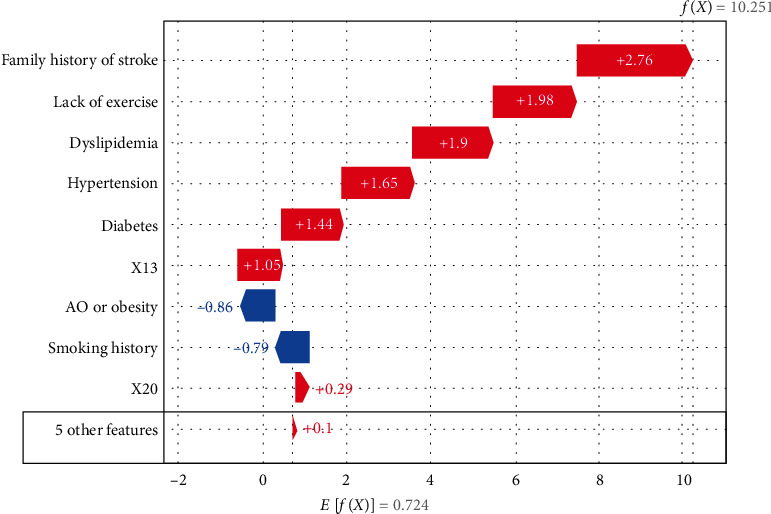
Importance ranking of characteristics of the first patient.

Here, we give an application of SHAP interpretable values in individual precision prediction and treatment guidance.  Figure 4 shows a waterfall diagram for a single patient with a factor vector (0, 0, 1, 0, 1, 1, 1, 0, 0, 1). At the bottom, *E*[*f*(*x*)] = 0.724 indicates the base value of shake of the overall sample. The bottom row represents five unimportant features, which have a positive impact of 0.1; *X*_20_ produces a 0.29 positive effect. Smoking history has a negative impact of 0.79, whereas *X*_13_ has a positive impact of 1.05, and family history of stroke has a positive impact of 2.76. Finally, the SHAP value for the first patient is 10.251 (shown in the upper right corner). Compared with the value of *E*(*x*), the value for this patient's illness is very large. Therefore, this individual meets the definition of a high-risk patient.

For this patient, family stroke history is the most important factor contributing to risk of stroke, followed by lack of exercise and dyslipidemia. If this individual develops hypertension and diabetes, the interaction of these factors with the others will aggravate the severity of the disease. The interaction between family stroke history and hypertension also plays an important part in the development of high stroke risk.

## 5. Conclusion and Discussion

In this study, risk factors were extracted and risk levels are predicted using stroke data from the Stroke Center of Lanzhou University Second Hospital from 2016 to 2018. First, risk factors were identified by sorting the importance of features. The results showed that the most important factors were hypertension, history of transient ischemia, history of stroke, and diabetes; family history of stroke, lack of exercise, dyslipidemia, smoking history, and apparent overweight or obesity were also factors with notable effects, whereas age and gender had negligible impact. Our results suggested that the XGBoost model was better at predicting stroke risk than other models according to almost all evaluation indices. Using Lundbery and Lee's optimal model and machine-learning-based SHAP, we could determine the impact of factors at each stroke level. Finally, we constructed a waterfall plot for a single patient to precisely show their level of stroke and the impact of different characteristics, to illustrate how the method could be used to guide accurate and personalized treatment for patients.

The study demonstrates precise prediction and identification of stroke level and the corresponding distinguishing features of a stroke patient. The proposed procedure involves a combination of feature selection, XGBoost classification, and SHAP interpretable analysis, which enables balancing of model accuracy and interpretability for medical applications in particular. The superiority of this approach has been demonstrated for personalized treatment of stroke patients. The XGBoost classifier can precisely determine the factors that distinguished each level of stroke in a patient group. Moreover, interpretation based on SHAP can give more precise information about the individual patient, which can help to guide individual diagnosis and stroke prevention strategies.

## Figures and Tables

**Table 1:** Description of the data.

Factor	2016			2017			2018		
	No.	Ratio	*P*	*N*	Ratio	*P*	*N*	Ratio	*P*
All	4296			3915			4180		
Stroke levels (*Y*)									
TIA: 0	88	2%		88	2.2%		100	2.4%	
Low risk: 1	3174	73.9%		2916	74.5%		2920	69.9%	
Medium risk: 2	446	10.4%		433	11.1%		491	11.7%	
High risk: 3	553	12.9%		442	11.3%		622	14.9%	
Stroke: 4	35	0.8%		36	0.9%		47	1.1%	
Continuous variable									
Age (*X*_1_)	4296		<0.01^∗∗^	3915		<0.01^∗∗^	4180		<0.01^∗∗^
	Mean	std		Mean	std		Mean	std	
TIA: 0	69.96	(12.34)		66.45	(11.23)		66.22	(11.31)	
Low risk: 1	60.39	(11.41)		56.94	(11.36)		58.10	(11.40)	
Medium risk:2	65.15	(11.75)		61.52	(11.73)		62.09	(11.66)	
High risk: 3	66.58	(10.30)		64.19	(9.93)		63.86	(10.26)	
Stroke: 4	68.00	(9.03)		66.17	(8.50)		64.98	(9.27)	
Discrete variables									
Gender (*X*_2_)			0.001^∗∗^			0.011^∗^			0.115
Female: 0	2240	52.1%		2013	51.4%		2138	51.1%	
Male: 1	2056	47.9%		1902	48.6%		2042	48.9%	
History of stroke (*X*_3_)			<0.01^∗∗^			<0.01^∗∗^			<0.01^∗∗^
Yes: 1	35	0.8%		36	0.9%		47	1.1%	
No: 0	4261	99.2%		3879	99.1%		4133	98.9%	
History of TIA (*X*_4_)			<0.01^∗∗^			<0.01^∗∗^			<0.01^∗∗^
Yes: 1	92	2.1%		91	2.3%		104	2.5%	
No: 0	4204	97.9%		3824	97.7%		4076	97.5%	
Family history of stroke (*X*_5_)			<0.01^∗∗^			<0.01^∗∗^			<0.01^∗∗^
Yes: 1	195	4.5%		177	4.5%		223	5.3%	
No: 0	4101	95.5%		3738	95.5%		3957	94.7%	
AF or VHD (*X*_6_)			<0.01^∗∗^			<0.01^∗∗^			<0.01^∗∗^
Yes: 1	112	2.6%		101	2.6%		119	2.8%	
No: 0	4184	97.4%		3814	97.4%		4061	97.2%	
Hypertension (*X*_7_)			<0.01^∗∗^			<0.01^∗∗^			<0.01^∗∗^
Yes: 1	684	15.9%		656	16.8%		938	22.4%	
No: 0	3612	84.1%		3259	83.2%		3242	77.6%	
Dyslipidemia (*X*_8_)			<0.01^∗∗^			<0.01^∗∗^			<0.01^∗∗^
Yes: 1	943	22%		1167	29.8%		1740	41.6%	
No: 0	3353	78%		2748	70.2%		2440	58.4%	
Diabetes (*X*_9_)			<0.01^∗∗^			<0.01^∗∗^			<0.01^∗∗^
Yes: 1	480	11.2%		409	10.4%		495	11.8%	
No: 0	3816	88.8%		3506	89.6%		3685	88.2%	
Smoking history (*X*_10_)			<0.01^∗∗^			<0.01^∗∗^			<0.01^∗∗^
Yes: 1	414	9.6%		291	7.4%		353	8.4%	
No: 0	3882	90.4%		3624	92.6%		3827	91.6%	
AO or obesity (*X*_11_)			<0.01^∗∗^			<0.01^∗∗^			<0.01^∗∗^
Yes: 1	679	15.8%		222	5.7%		245	5.9%	
No: 0	3617	84.2%		3693	94.3%		3935	94.1%	
Lack of exercise (*X*_12_)			<0.01^∗∗^			<0.01^∗∗^			<0.01^∗∗^
Yes: 1	524	12.2%		444	11.3%		556	13.3%	
No: 0	3772	87.8%		3471	88.7%		3624	86.7%	

TIA: transient ischemic attack; AF: atrial fibrillation; VHD: valvular heart disease; AO: apparently overweight. Significance analyses were performed by analysis of variance. All tests were two-sided. ^∗^Statistically significant *P* values (*P*<0.05); ^∗∗^statistically very significant *P* values (*P* < 0.01).

**Table 2:** Correlations and NMI values for the data.

Factor	2016		2017		2018	
	*ρ*(*X*_*i*_, *Y*)	NMI(*X*_*i*_, *Y*)	*ρ*(*X*_*i*_, *Y*)	NMI(*X*_*i*_, *Y*)	*ρ*(*X*_*i*_, *Y*)	NMI(*X*_*i*_, *Y*)
Age (*X*_1_)	0.1710	0.0284	0.1894	0.0312	0.1650	0.0270
Gender (*X*_2_)	-0.0165	0.0034	-0.0020	0.0028	0.0101	0.0020
History of stroke (*X*_3_)	0.2021	0.1064	0.2163	0.1175	0.2258	0.1249
History of TI (*X*_4_)	-0.2973	0.2129	-0.3191	0.2274	-0.3080	0.2173
Family history of stroke (*X*_5_)	0.3933	0.1722	0.3832	0.1583	0.3718	0.1390
AF or VHD (*X*_6_)	0.2253	0.0814	0.2208	0.0863	0.2151	0.0711
Hypertension (*X*_7_)	0.7232	0.4331	0.7623	0.4772	0.7926	0.5196
Dyslipidemia (*X*_8_)	0.2538	0.0858	0.1974	0.0764	0.1409	0.0668
Diabetes (*X*_9_)	0.5057	0.2921	0.4848	0.2834	0.4817	0.2605
Smoking history (*X*_10_)	0.2469	0.0717	0.1870	0.0553	0.2310	0.0644
AO or obesity (*X*_11_)	0.1630	0.0513	0.1321	0.0337	0.1569	0.0351
Lack of exercise (*X*_12_)	0.3247	0.0291	0.2957	0.1558	0.3531	0.1551

*ρ*(*X*_*i*_, *Y*) represents the Spearman correlation between *X*_*i*_ and *Y* (1 = 2, 3, ⋯, 12).*ρ*(*X*_*i*_, *Y*) represents the Pearson correlation between *X*_1_.

**Table 3:** Performance evaluation using fivefold cross-validation for different models: mean (standard deviation).

Model	AUC	Accuracy	*F*_1_-macro	Recall-macro	Precision-macro
MLR	0.9931 (0.0006)	0.9456 (0.0061)	0.9587 (0.0039)	0.9895 (0.0021)	0.9362 (0.0054)
MCSVM	0.9801 (0.0019)	0.9723 (0.0021)	0.9751 (0.0019)	0.9766 (0.0024)	0.9736 (0.0021)
XGBoost	0.9999 (0.0000)	0.9927 (0.0015)	0.9929 (0.0020)	0.9942 (0.0039)	0.9918 (0.0018)
LightGBM	0.9998 (0.0000)	0.9916 (0.0028)	0.9924 (0.0022)	0.9918 (0.0044)	0.9930 (0.0017)

**Table 4:** Comparisons of AUC values for the forward stepwise method with interactive effects for different models.

Algorithm	*M*_0_: original	*M*_1_ : *M*_0_ + *X*_13_	*M*_2_ : *M*_1_ + *X*_14_	*M*_3_ : *M*_2_ + *X*_20_	*M*_4_ : *M*_3_ + *X*_17_
MLR	0.9931	0.9993 (+0.0062)	0.9997 (+0.0004)	0.9997 (+0.0000)	1 (+0.0003)
MCSVM	0.9801	0.9983 (+0.0182)	0.9991 (+0.0008)	0.9987 (−0.0004)	1 (+0.0013)
XGBoost	0.9999	0.9999	0.9999	0.9999	0.9999
LightGBM	0.9999	0.9999	0.9999	0.9999	0.9999

## Data Availability

Data are available upon reasonable request.
